# Author Correction: Extracting backbones in weighted modular complex networks

**DOI:** 10.1038/s41598-021-88213-8

**Published:** 2021-04-20

**Authors:** Zakariya Ghalmane, Chantal Cherifi, Hocine Cherifi, Mohammed El Hassouni

**Affiliations:** 1 LRIT, URAC No 29, Rabat IT Center, Mohammed V University in Rabat, Rabat, Morocco; 2 DISP Laboratory, University of Lyon 2, Lyon, France; 3 LIB EA 7534, University of Burgundy, Dijon, France; 4 FLSH, Mohammed V University in Rabat, Rabat, Morocco

Correction to: *Scientific Reports* 10.1038/s41598-020-71876-0, published online 23 September 2020

The original version of this Article contained an error in Affiliation 1 and 4, which were incorrectly given as ‘LRIT, URAC No 29, Rabat IT Center, Mohammed V University, Rabat, Morocco ‘and ‘FLSH, Mohammed V University, 10500 Rabat, Morocco’.

The correct affiliations are listed below:

Affiliation 1

LRIT, URAC No 29, Rabat IT Center, Mohammed V University in Rabat, Rabat, Morocco

Affiliation 4

FLSH, Mohammed V University in Rabat, Rabat, Morocco

